# Comparison of accuracy of maxilla between virtual surgical planning and conventional surgical planning in bimaxillary orthognathic surgery: a randomized controlled trial

**DOI:** 10.1186/s40902-025-00469-6

**Published:** 2025-07-03

**Authors:** Loi Phuoc Nguyen, Chon Thanh Ho Nguyen, Tuan Van Nguyen, Hai Tien Do, Chanh Trung Le, Jun-Young Kim

**Affiliations:** 1 Department of Maxillofacial Surgery, Faculty of Odonto-Stomatology, University of Medicine and Pharmacy at Ho Chi Minh City, Ho Chi Minh City, Viet Nam; 2 National Hospital of Odonto-Stomatology in Ho Chi Minh City, Ho Chi Minh City, Viet Nam; 3 Department of Oral & Maxillofacial Surgery, Yonsei University College of Dentistry, Seoul, Republic of Korea; 4 Institute for Innovation in Digital Healthcare, Yonsei University, Seoul, Republic of Korea

**Keywords:** Virtual surgical planning, Orthognathic surgery, Maxillary accuracy, 3D planning, Randomized controlled trial

## Abstract

**Background:**

Virtual surgical planning (VSP) improves accuracy in orthognathic surgery, but its differences from conventional surgical planning (CSP) remain unclear. This study compares VSP and CSP accuracy in maxillary repositioning.

**Methods:**

A randomized controlled trial of 20 patients undergoing bimaxillary surgery was conducted. Patients were assigned to VSP (3D planning, 3D-printed splints) or CSP (cast model surgery, conventional splints). Pre- and postoperative Computed Tomography (CT) scans were superimposed using voxel-based registration, measuring anteroposterior (Y), mediolateral (X), and vertical (Z) positional changes of A point, ANS, U1, U3, U6 landmarks.

**Results:**

No significant differences in planned and actual surgical outcomes (*p* > 0.05). 2D planning (P2D) and 3D planning (P3D) showed significant differences in key maxillary landmarks, indicating that 3D planning provides additional refinements in skeletal positioning. However, VSP showed larger absolute discrepancies in U1L, U1R, U3L, U6L (*p* < 0.05), particularly in the anteroposterior (Y-axis) direction. Splint thickness and condylar simulation methods could also affect accuracy.

**Conclusions:**

VSP and CSP provide comparable accuracy; however, VSP shows greater anterior–posterior discrepancies. Further studies should examine splint design and condylar modeling to optimize surgical precision.

## Introduction

Orthognathic surgery is a widely used procedure for correcting dentofacial deformities, significantly improving both functional and aesthetic aspects of the patients. This surgical intervention is commonly performed to treat conditions such as malocclusion, facial asymmetry, and obstructive sleep apnea, enhancing mastication efficiency, pronunciation, and overall facial harmony [4]. Orthognathic surgery changes the maxilla and mandible position to restore both occlusion and facial proportions, leading to higher quality of life for patients.

Besides the surgical skill required to perform orthognathic procedures, precise preoperative planning is crucial to achieving optimal outcomes. Traditional planning methods, such as two-dimensional (2D) cephalometric analysis and cast model surgery, often cause some errors due to manual handling and the inability to capture the complexity of three-dimensional structures [2, 4]. Minor errors over many steps can lead to inaccuracy in predicting postoperative maxillary and mandibular position. Accurate planning is critical to minimizing postoperative complications and achieving the desired functional and aesthetic goals.

Today, virtual surgical planning (VSP) has become an indispensable tool in orthognathic surgery, offering greater precision and predictability. With the integration of CT imaging data, VSP provides detailed three-dimensional visualization of skeletal structures, allowing for more accurate identification of anatomical landmarks, assessment of bone morphology, and detection of asymmetries [1, 14]. This advanced imaging enhances precise osteotomy planning and accurate simulation of surgical movements, ultimately improving surgical outcomes and reducing intraoperative adjustments. This approach improves communication between the surgical team and the patient while facilitating intraoperative execution with reduced reliance on intraoperative adjustments.

Although there are numerous studies [4, 5, 8, 10, 12, 15, 18] which have compared the accuracy of VSP and conventional surgical planning (CSP), none have clearly identified at which stage these differences arise. It remains unclear whether discrepancies caused by preoperative planning, 2D analysis, cast model surgery, splints or intraoperative execution. Understanding these distinctions is critical to refining orthognathic surgery planning, techniques and improving clinical outcomes.

Therefore, we conducted a randomized controlled trial (RCT) to compare these VSP and CSP, aiming to determine which phase contributes to the differences in accuracy between the two methods. The null hypothesis was that there would be no meaningful difference between VSP and CSP in maxillary movements. To evaluate this, we superimposed preoperative and postoperative CT images, differences in the anteroposterior, mediolateral, and superoinferior changes of the maxillary landmarks were calculated to assess accuracy and validate the effectiveness of virtual surgical planning.

## Materials and methods

### Study design

This study was conducted as a prospective, single-center, randomized blinded case-controlled trial. This research protocol was reviewed and approved by the Ethics Committee of the University of Medicine and Pharmacy at Ho Chi Minh city with registration number 647/HĐĐĐ-ĐHYD and registered at the ClinicalTrials.gov (registration number NCT06940024).

All participants were fully informed about their management options and signed a consent agreement form before enrolment in the study. The trial adhered to ethical guidelines for clinical research, ensuring patients rights, safety, and confidentiality were maintained throughout the study period.

### Study subjects

This study was performed in the National Hospital of Odonto – Stomatology in Ho Chi Minh city from August 2023 to February 2025. Patients had aged between 18 to 30 years with diagnosed malocclusion requiring orthognathic surgery were included in this research. Patients were excluded on the following criteria: (1) cleft lip and palate congenital abnormalities; (2) the facial deformities were caused by trauma, tumor, or iatrogenic factors; (3) temporomandibular joint disorders; (4) history of previous orthognathic surgery; (5) patients scheduled for multipiece Le Fort I osteotomy.

A total of 20 patients me the inclusion criteria and agreed to participate. All patients had completed presurgical orthodontic treatment before undergoing surgical management.

#### Randomization and blinding

Each patient underwent both 2D lateral cephalometric analysis and 3D virtual surgical planning prior to surgery. Additionally, both a traditional surgical splint and a 3D-printed surgical splint were prepared for each patient.

During surgery, an operating room nurse performed a random draw to determine which surgical guide would be used for each patient. The allocation was recorded accordingly. The researchers were blinded to the guide selection, knowing only that patients were assigned to either Group 1 or Group 2, without knowledge of which group was the test or control.

The group identities (VSP or CSP) were only revealed after all data analysis was completed to maintain the integrity of the randomized controlled trial.

Patients were randomly assigned into two groups:

- Test group (VSP): Surgical planning was conducted using three-dimensional (3D) imaging, virtual osteotomy simulations, and 3D-printed surgical splints.
- Control group (CSP): three-dimensional (3D) imaging, virtual osteotomy simulations, cast model surgery, and resin occlusal surgical splints were used for planning.

## Methods

### Preoperative examination and surgical planning

All patients underwent comprehensive preoperative examination and planning, which included clinical examination, radiographic imaging (panoramic, lateral cephalometric, posteroanterior cephalometric, and CT scans), 2D cephalometric analysis, photography, and dental impressions.

**Fig. 1:**
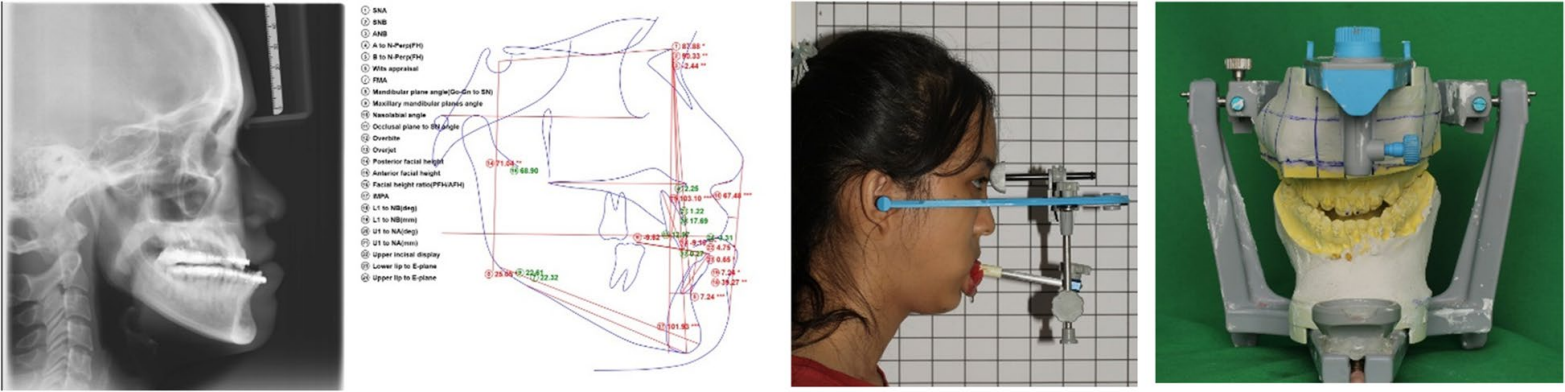
Preoperative examination and surgical planning

Each patient underwent facebow registration, intermaxillary relationship assessment, and semi-adjustable articulator mounting in centric relation using the standard Frankfort horizontal plane (Fig. 1). The final occlusion was determined by an orthodontist, ensuring proper two jaw relation preoperative. Patient dental models were scanned using the Autoscan-DS-EX Pro scanner (Shining 3D).

The surgical planning was based primarily on clinical findings and 2D cephalometric analysis using WebCeph software. The 2D cephalometric surgical parameters included maxillary dental midline positioning, anteroposterior and vertical positioning of the maxillary incisors, and maxillary occlusal plane canting adjustment using the canine and first molar references.

### 3D Simulation

**Fig. 2:**
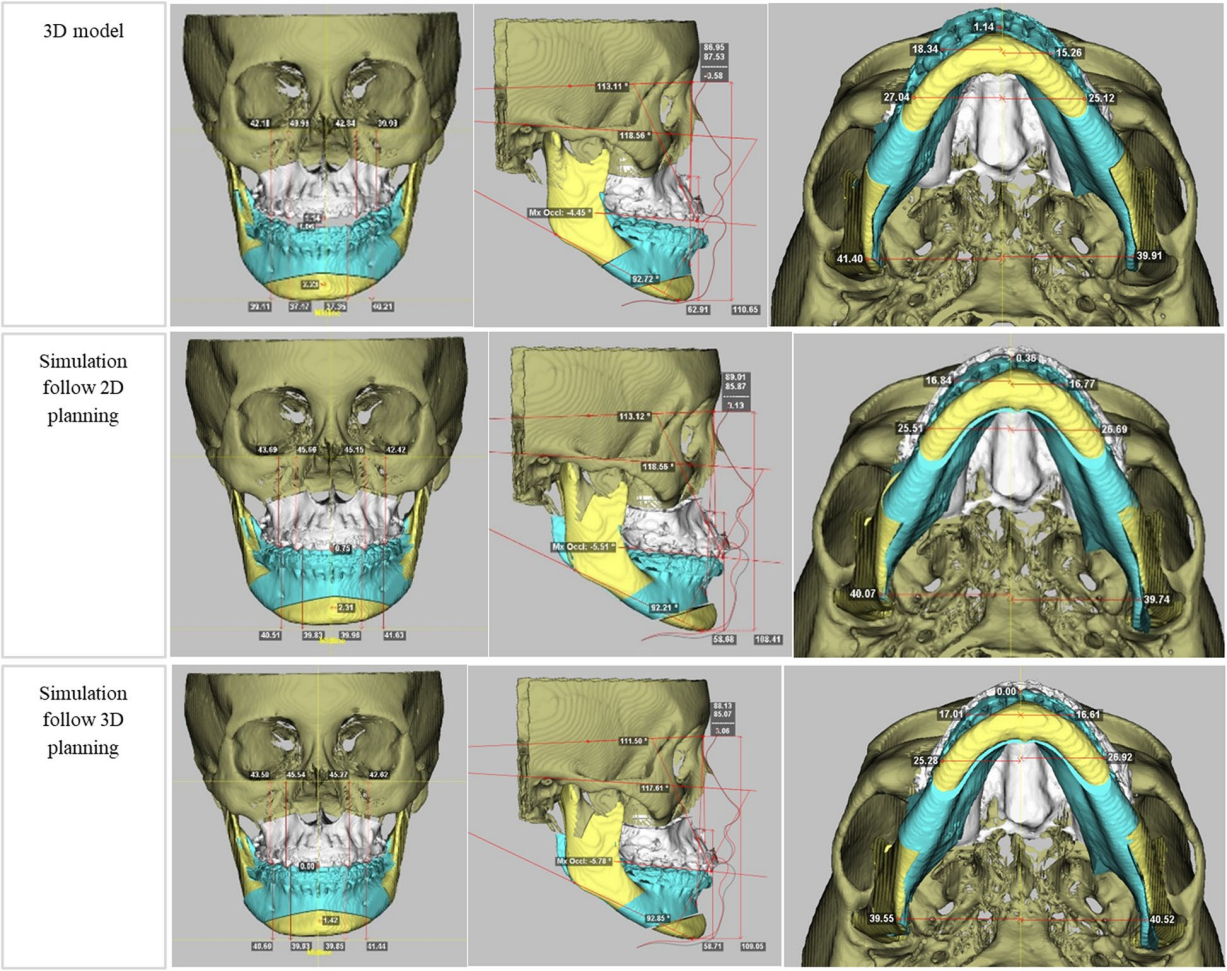
Skull model preoperative; Simulate two-jaw movements follow 2D planning; Simulate maxillomandibular complex position by following 3D step guides

A 3D skull model was constructed using anatomical reference planes to ensure accurate orientation: the horizontal plane (passing through the Nasion points and parallel to the Frankfort horizontal plane), the midsagittal plane (passing through the Nasion and Basion perpendicular to the Frankfort horizontal plane), and the coronal plane (passing through the Nasion perpendicular to both the horizontal and midsagittal planes) (Table 1 and Fig. 2).

**Table 1:** Descriptions of the landmark points and reference planes

Landmark	Description
Landmark points	
Nasion (Na)	The most anterior point at the junction of the nasal and frontal bones in the mid-sagittal plane
Porion (Po)	The most superior at the bone surface of the external auditory meatus
Orbitale (Or)	The most inferior and anterior point of the infraorbital edge
Basion (Ba)	The most inferior-posterior point on the anterior margin of the foramen magnum
A	The deepest point on the curvature of the maxillary alveolar process
ANS	The anterior point of the nasal floor
U1	The maxillary central incisors midpoint
U1L	The maxillary left central incisor tip
U1R	The maxillary right central incisor tip
U3L	The maxillary left canine tip
U3R	The maxillary right canine tip
U6L	The mesiobuccal cusp tip of the left maxillary first molar
U6R	The mesiobuccal cusp tip of the right maxillary first molar
Reference planes	
Frankfort (FH) plane	The plane passing through four points, including the bilateral Porions and Orbitales
Mid-sagittal plane	The perpendicular plane to the FH plane and passing through the Nasion point and the Basion point
Coronal plane	The plane perpendicular to both the FH plane and the mid-sagittal plane, passing through the Nasion point

The mandible was repositioned to its final occlusion, as determined in the preoperative planning. The maxillomandibular complex was subsequently adjusted based on 2D cephalometric planning parameters including upper jaw dental midline, maxillary canting, horizontal and vertical position of maxillary central incisors midpoint. The maxillary landmark positions (A point, ANS, U1, U1L, U1R, U3L, U3R, U6L, U6R) were recorded as 2D planning (P2D) (Table 1 and Fig. 2).

Next, 3D virtual surgical planning (VSP) refinements were applied to optimize occlusal plane canting, midline discrepancies, yaw rotation for maxillary and mandibular symmetry, and precise anteroposterior and vertical positioning of the maxillary incisors and first molars, based on normative Vietnamese population standards. After these adjustments, the final maxillary landmark positions were then recorded as 3D planning (P3D) (Fig. 2).

### Comparison between P2D and P3D

The differences in landmark movement distances (A point, ANS, U1, U1L, U1R, U3L, U3R, U6L, U6R) between P2D and P3D were analyzed to assess discrepancies between 2 and 3D planning approaches.

### Group allocation

**Fig. 3:**
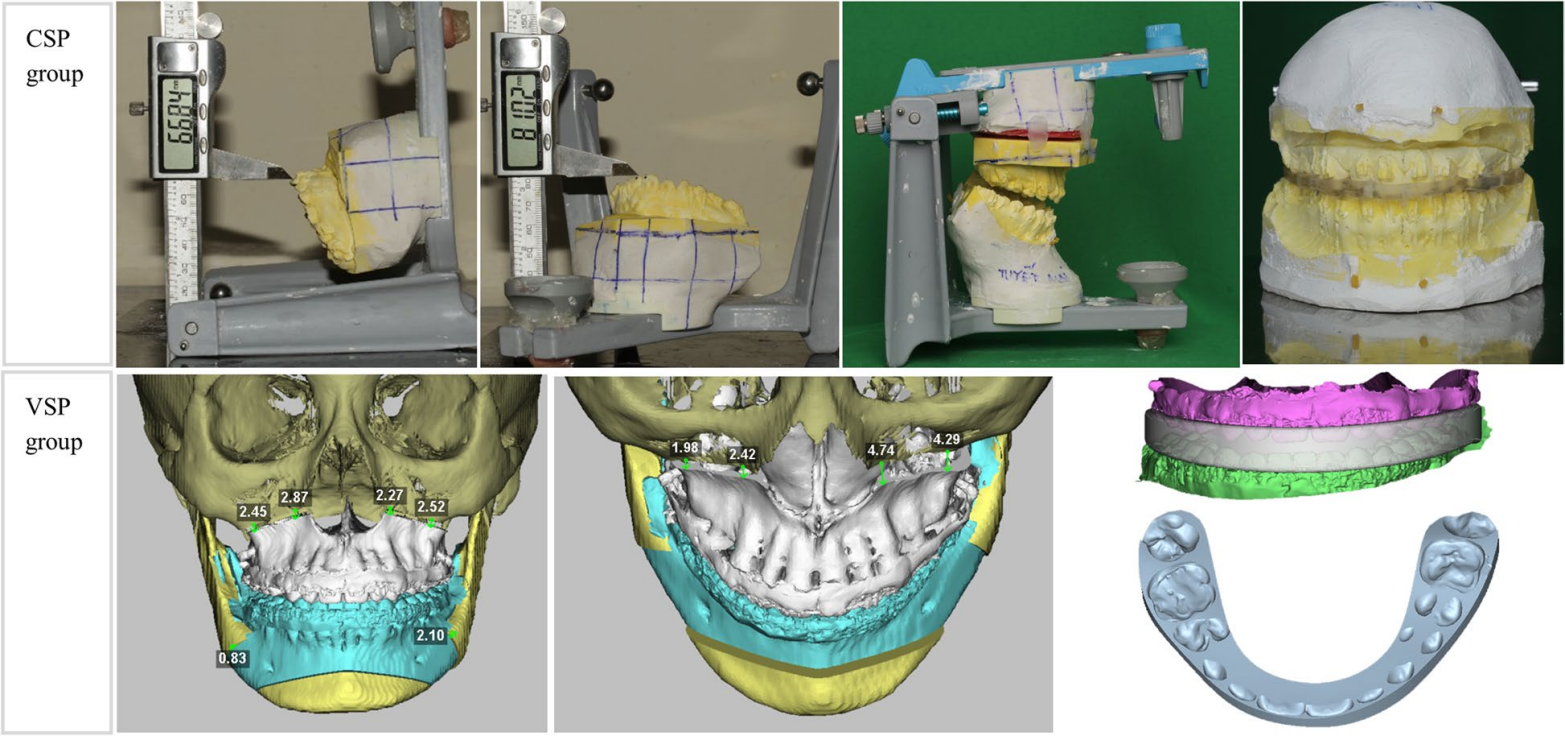
Steps of model cast surgery; simulation of maxillary postion and splint design

After determining the desired the maxillary and mandibular position by 3D planning, involved fabricating surgical splints using two different methods:

- CSP Group (Conventional Resin Occlusal Splint): Splints were manually fabricated based on P3D movement values. Maxillary landmark points were marked, and the upper jaw cast model was segmented and repositioned to match P3D maxillary to the nearest approximation (Fig. 3) base on anterior–posterior and superior-inferior changes of these points. The actual movement distances of U1L, U1R, U3L, U3R, U6L, and U6R were recorded as planning cast model (PCM).
- VSP Group (Virtual Digital Occlusal 3D Print Splint): Digital surgical splints were generated using Dolphin software and a Form 3D printer (Fig. 3).

### Surgical procedures

All patients underwent LeFort I osteotomy and bilateral sagittal split osteotomy (BSSO), performed by an experienced maxillofacial surgeon using a maxilla-first approach. The surgical sequence and fixation protocol were standardized across all cases to ensure consistency and accuracy in skeletal positioning.

Maxillary osteotomy was initially performed using a LeFort I approach, facilitating mobilization of the maxilla. Subsequently, the maxilla was positioned according to the preoperative 3D surgical plan using the intermediate splint. The maxillary position was carefully compared with the simulation images of the planned maxillary movement (Fig. 3) to ensure accuracy. Following temporary fixation of the maxilla, rigid fixation was achieved using four mini plates and screws.

Subsequent to maxillary fixation, the mandibular osteotomy was performed using a BSSO technique. The mandibular segments were meticulously mobilized and repositioned using the final surgical splint to establish the planned occlusion and skeletal harmony. Each hemimandible was fixed using two miniplates and screws, ensuring stability while allowing for controlled postoperative healing.

Once accuracy was confirmed, soft tissues were reapproximated and sutured in layers to facilitate optimal healing. Intermaxillary fixation (IMF) was not routinely applied, but light elastics were used as necessary to guide postoperative occlusion.

### Postoperative skeletal accuracy analysis

Two weeks after surgery, CT imaging was performed on all patients in occlusion with the final splint, prior to postoperative orthodontic treatment.

**Fig. 4:**
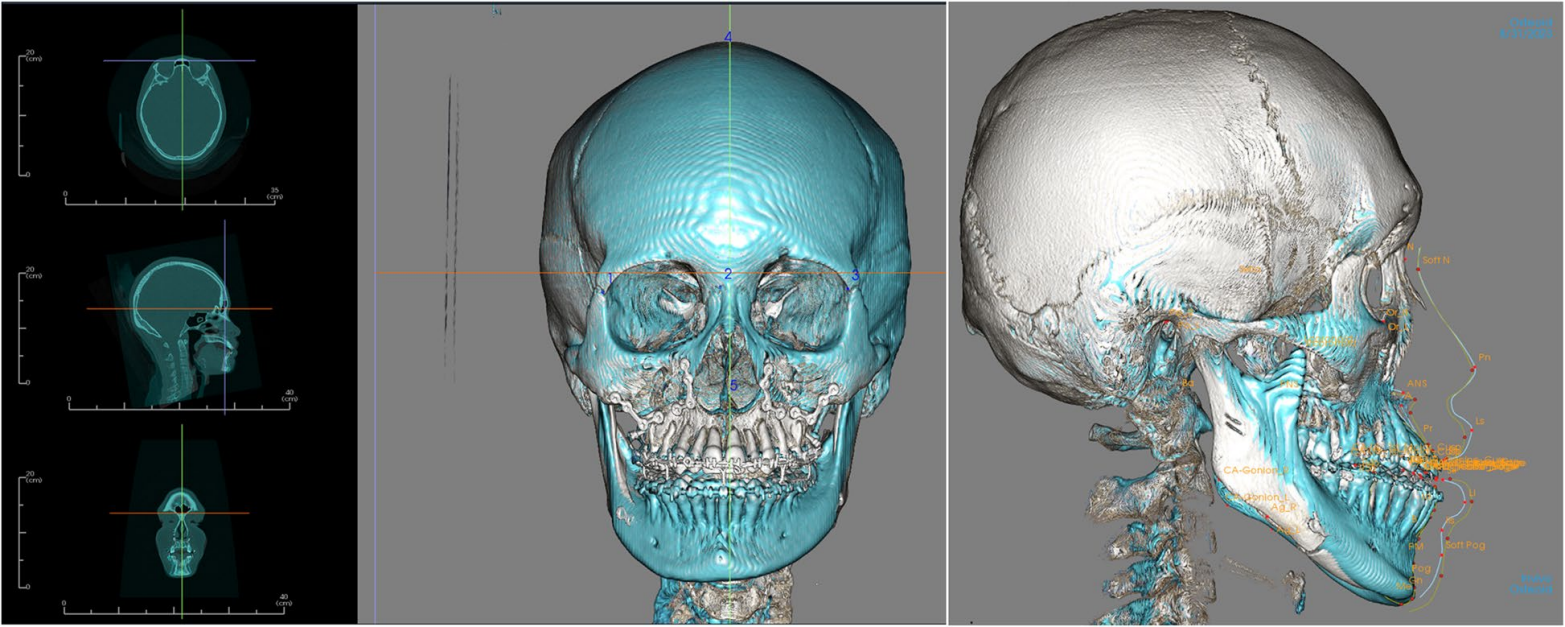
Superimposition of skull model preoperative and postoperative based on non surgical cranial

The preoperative and postoperative CT scans were superimposed using Invivo 7.0 software (Anatomage, San Jose, CA). To ensure precise alignment, the superimposition was performed on non-surgical cranial reference areas using voxel-based registration. The reference planes used for alignment were as previously described (Fig. 4):

- X-axis (Medio-Lateral movements): Positive values indicate movement toward the left.
- Y-axis (Anterior–Posterior movements): Positive values indicate forward movement.
- Z-axis (Superior-Inferior movements): Positive values indicate downward movement.

The actual movement distances of A point, ANS, U1, U1L, U1R, U3L, U3R, U6L, and U6R were recorded as Actual values.

The accuracy of surgical outcomes was evaluated through:

- Comparison within each group – The differences between planned surgical position and actual postoperative position were analyzed to assess the accuracy of maxillary repositioning.
- Comparison between VSP and CSP groups – Discrepancies between planned and actual movements were compared between VSP and CSP to determine which planning method provided superior accuracy.

### Measurement methods for accuracy assessment

The intraclass correlation coefficient (ICC) was used to assess measurement reliability. A subset of 10 patients was randomly selected, and measurements were repeated after two weeks to evaluate consistency. The ICC was used to quantify intra-examiner reliability, resulting in an excellent outcome (average ICC value: 0.996; 95% CI, 0.996–0.997).

### Statistical analysis

A normality test was performed to determine the appropriate statistical approach:

- If data followed a normal distribution, comparisons between planned and actual outcomes were performed using a paired t-test, while differences between VSP and CSP groups were analyzed using an independent t-test.
- If data were not normally distributed, Wilcoxon signed-rank tests were used for paired comparisons, and Mann–Whitney U tests were applied for group comparisons.

A *p*-value < 0.05 was considered statistically significant.

## Results

The study included two groups: the test group (VSP) and the control group (CSP). The demographic analysis showed no significant differences between the two groups. The mean age of the VSP group was 22.4 ± 2.37 years, while the CSP group had a mean age of 24.30 ± 4.14 years (*p* = 0.23). The gender distribution was similar, with a slightly higher proportion of females in both groups. Class III malocclusion was the most common deformity in both groups (9 cases in VSP vs. 8 cases in CSP) (Table 2).

**Table 2:** Baseline characteristics of the study population

Description	VSP	CSP	Total
Age, year			
Mean	22.4 ± 2.37	24.30 ± 4.14	23.35 ± 3.42
Range	20—28	20—30	20—30
p	0.23^a^		
Sex			
Male	3	2	5
Female	7	8	15
Deformiaty diagnosis			
Class II	1	2	3
Class III	9	8	17
Cephalometric analysis	VSP	CSP	p
SNA	83.73 ± 2.88	83.97 ± 3.57	0.87
SNB	85.81 ± 3.90	83.39 ± 7.30	0.37
ANB	−2.08 ± 2.78	0.58 ± 4.56	0.13
A to N-Perp(FH)	1.61 ± 2.33	1.34 ± 2.44	0.80
B to N-Perp(FH)	6.20 ± 4.05	1.25 ± 9.75	0.16
FMA	26.52 ± 4.24	29.21 ± 4.95	0.21
Mandibular plane angle(Go-Gn to SN)	32.78 ± 5.99	34.78 ± 6.74	0.49
Maxillary mandibular planes angle	25.64 ± 3.96	27.53 ± 4.66	0.34
Nasolabial angle	83.58 ± 12.15	85.37 ± 8.26	0.71
Occlusal plane to SN angle	16.34 ± 3.88	20.48 ± 7.89	0.15
Wits appraisal	−10.61 ± 3.56	−9.45 ± 7.58	0.67
Overjet	−4.77 ± 3.37	−3.24 ± 6.09	0.50
Overbite	1.51 ± 1.73	0.88 ± 2.56	0.53
Posterior facial height	76.34 ± 6.53	75.01 ± 5.12	0.62
Anterior facial height	119.38 ± 11.65	118.62 ± 7.30	0.86
Facial height ratio(PFH/AFH)	64.13 ± 4.23	63.34 ± 4.35	0.68
IMPA	88.03 ± 9.09	92.19 ± 4.66	0.21
U1 to NA(mm)	5.13 ± 2.60	4.37 ± 2.26	0.50
U1 to NA(deg)	25.97 ± 5.77	21.10 ± 5.96	0.08
Upper incisal display	4.17 ± 2.08	3.95 ± 2.03	0.81

^a^All data are expressed as mean ± SD. We performed the independent t-test

The cephalometric analysis indicated no statistically significant differences (*p* > 0.05) in most parameters, demonstrating baseline similarity between the VSP and CSP groups. These findings confirm that both groups had comparable skeletal and occlusal characteristics preoperatively, allowing for an objective comparison of postoperative outcomes.

### 2D planning vs 3D planning vs actual movements after surgery

The comparison of virtual surgical planning (VSP) and conventional surgical planning (CSP) in bimaxillary orthognathic surgery revealed variations in positional accuracy across the X, Y, and Z axes.

The comparison between 2D planning, 3D planning, and actual postoperative outcomes was analyzed across the X (horizontal), Y (anterior–posterior), and Z (vertical) axes. For the horizontal (X) axis and vertical (Z) axis, no significant differences were found between 2 and 3D planning across all landmarks (*p* > 0.05), indicating similar positioning accuracy in both methods. A significant difference was observed between the 2D and 3D planning groups at several landmarks, including U1L (*p* = 0.028), U1R (*p* = 0.046), U3L (*p* = 0.040), U3R (*p* = 0.039), and U6R (*p* = 0.035) in the anterior–posterior (Y) axis (Table 3).

**Table 3:** Comparison of positional changes in maxillary landmarks between P2D, P3D, and actual postoperative outcomes

Point	X – axis					Y – axis					Z – axis				
	P2D	P3D	Actual	p*	p**	P2D	P3D	Actual	p*	p**	P2D	P3D	Actual	*p* *	*p* **
A	0.14 ± 1.09	0.05 ± 0.93	−0.17 ± 0.73	0.572	0.43	1.53 ± 2.86	1.42 ± 2.92	1.23 ± 2.47	0.101	0.50	−0.53 ± 2.63	−0.39 ± 2.65	−0.14 ± 3.36	0.201	0.37
ANS	0.12 ± 1.14	0.00 ± 1.09	−0.16 ± 0.72	0.482	0.59	1.61 ± 3.04	1.47 ± 3.10	0.58 ± 2.48	0.094	0.09	−0.50 ± 2.69	−0.36 ± 2.71	0.11 ± 3.04	0.239	0.10
U3L	0.26 ± 1.07	0.26 ± 0.84	−0.26 ± 1.28	0.975	0.12	1.37 ± 2.36	1.74 ± 2.62	1.63 ± 2.70	0.040	0.79	−0.55 ± 2.73	−0.44 ± 2.86	−0.61 ± 3.22	0.188	0.47
U3R	0.24 ± 1.08	0.19 ± 0.86	−0.32 ± 1.59	0.732	0.16	1.32 ± 2.38	0.93 ± 2.38	0.96 ± 2.46	0.039	0.92	−0.50 ± 2.29	−0.36 ± 2.32	−0.40 ± 3.06	0.378	0.89
U6L	0.26 ± 0.99	0.55 ± 0.77	−0.04 ± 1.08	0.248	0.09	1.41 ± 2.44	1.92 ± 2.87	1.58 ± 2.65	0.051	0.41	−0.78 ± 2.50	−0.53 ± 2.71	−0.49 ± 2.71	0.077	0.87
U6R	0.24 ± 1.01	0.56 ± 0.85	0.54 ± 1.16	0.233	0.97	1.34 ± 2.47	0.71 ± 2.57	1.03 ± 2.58	0.035	0.47	−0.73 ± 1.75	−0.41 ± 2.02	−0.16 ± 2.32	0.236	0.44
U1L	0.25 ± 1.12	0.04 ± 1.08	−0.13 ± 1.45	0.183	0.59	1.33 ± 2.33	1.42 ± 2.37	1.39 ± 2.68	0.028	0.92	−0.41 ± 2.83	−0.35 ± 2.86	−0.57 ± 3.51	0.136	0.35
U1R	0.25 ± 1.12	0.03 ± 1.08	−0.23 ± 1.53	0.170	0.44	1.32 ± 2.33	1.23 ± 2.30	1.22 ± 2.59	0.046	0.98	−0.39 ± 2.74	−0.32 ± 2.74	−0.41 ± 3.39	0.332	0.71
U1	0.25 ± 1.12	0.03 ± 1.08	−0.20 ± 1.48	0.176	0.48	1.33 ± 2.33	1.33 ± 2.33	1.30 ± 2.64	0.813	0.94	−0.40 ± 2.78	−0.34 ± 2.80	−0.49 ± 3.44	0.226	0.52

All data are expressed as mean ± SD. We performed the pair t-test and Wilcoxon test

^*^ *p* value between P2D and P3D

^**^ *p* value between P3D and actual results postoperation

However, comparison of 3D planning (VSP) with the actual postoperative outcomes, revealed no statistically significant differences (*p* > 0.05).

### Deviation between simulation (3D) and actual postoperation

The comparison between virtual surgical planning (VSP) simulation and actual postoperative outcomes showed no statistically significant differences across the horizontal (X), anterior–posterior (Y), and vertical (Z) axes (*p* > 0.05) (Table 4).

**Table 4:** Comparison of positional changes in maxillary landmarks between P3D and actual postoperative outcomes in each group and differences between the two groups

Direction		VSP			CSP			Different		
		P3D	Post op	p	P3D	Post op	p	Test	Control	p
X	A	0.09 ± 1.06	0.07 ± 0.58	0.97	0.02 ± 0.84	−0.42 ± 0.81	0.31	−0.01 ± 1.26	−0.44 ± 1.27	0.47
	ANS	0.14 ± 1.24	0.09 ± 0.56	0.91	−0.13 ± 0.98	−0.41 ± 0.80	0.53	−0.05 ± 1.38	−0.28 ± 1.36	0.71
	U3L	0.00 ± 1.00	−0.44 ± 0.76	0.17	0.51 ± 0.61	−0.08 ± 1.67	0.33	−0.44 ± 0.93	−0.59 ± 1.80	0.83
	U3R	−0.07 ± 0.99	−0.54 ± 1.47	0.23	0.45 ± 0.66	−0.10 ± 1.74	0.39	−0.47 ± 1.16	−0.55 ± 1.92	0.91
	U6L	0.40 ± 0.79	0.02 ± 0.87	0.26	0.71 ± 0.76	−0.11 ± 1.30	0.20	−0.38 ± 1.00	−0.81 ± 1.86	0.52
	U6R	0.37 ± 0.83	0.93 ± 0.87	0.22	0.75 ± 0.87	0.16 ± 1.33	0.34	0.56 ± 1.35	−0.59 ± 1.84	0.13
	U1L	−0.29 ± 1.16	−0.24 ± 0.89	0.89	0.37 ± 0.94	−0.03 ± 1.90	0.49	0.05 ± 1.05	−0.39 ± 1.75	0.50
	U1R	−0.29 ± 1.15	−0.37 ± 0.93	0.78	0.35 ± 0.95	−0.09 ± 2.01	0.48	−0.08 ± 0.91	−0.44 ± 1.91	0.60
	U1	−0.29 ± 1.16	−0.31 ± 0.87	0.95	0.36 ± 0.94	−0.09 ± 1.95	0.46	−0.02 ± 0.97	−0.45 ± 1.83	0.52
Y	A	1.61 ± 2.92	1.15 ± 2.70	0.34	1.23 ± 3.06	1.30 ± 2.36	0.86	−0.46 ± 1.46	0.07 ± 1.14	0.38
	ANS	1.71 ± 3.08	0.41 ± 2.35	0.12	1.24 ± 3.27	0.76 ± 2.72	0.49	−1.30 ± 2.38	−0.48 ± 2.09	0.42
	U3L	1.86 ± 2.64	1.21 ± 2.82	0.39	1.62 ± 2.73	2.04 ± 2.66	0.28	−0.65 ± 2.31	0.42 ± 1.16	0.20
	U3R	0.93 ± 2.39	0.70 ± 2.69	0.73	0.93 ± 2.50	1.23 ± 2.33	0.37	−0.23 ± 2.00	0.30 ± 1.03	0.47
	U6L	2.11 ± 2.79	1.33 ± 2.59	0.24	1.73 ± 3.09	1.83 ± 2.83	0.85	−0.78 ± 1.98	0.10 ± 1.59	0.29
	U6R	0.77 ± 2.54	1.10 ± 2.79	0.67	0.66 ± 2.75	0.96 ± 2.49	0.52	0.33 ± 2.41	0.30 ± 1.43	0.97
	U1L	1.47 ± 2.43	1.06 ± 2.89	0.56	1.38 ± 2.44	1.71 ± 2.56	0.47	−0.41 ± 2.16	0.33 ± 1.38	0.37
	U1R	1.24 ± 2.36	0.85 ± 2.82	0.56	1.21 ± 2.37	1.59 ± 2.44	0.39	−0.39 ± 2.06	0.38 ± 1.31	0.33
	U1	1.36 ± 2.39	0.96 ± 2.86	0.57	1.30 ± 2.39	1.63 ± 2.50	0.45	−0.40 ± 2.12	0.33 ± 1.34	0.37
Z	A	0.22 ± 2.15	0.47 ± 3.08	0.56	−0.99 ± 3.07	−0.75 ± 3.67	0.51	0.25 ± 1.32	0.24 ± 1.12	0.99
	ANS	0.25 ± 2.19	0.65 ± 2.64	0.34	−0.97 ± 3.13	−0.42 ± 3.46	0.19	0.40 ± 1.25	0.55 ± 1.22	0.79
	U3L	0.35 ± 2.44	0.18 ± 2.55	0.63	−1.23 ± 3.14	−1.39 ± 3.75	0.62	−0.17 ± 1.08	−0.16 ± 0.98	0.98
	U3R	−0.09 ± 1.83	−0.19 ± 2.38	0.80	−0.62 ± 2.81	−0.61 ± 3.73	0.98	−0.10 ± 1.20	0.01 ± 1.54	0.86
	U6L	0.25 ± 2.51	0.01 ± 2.18	0.51	−1.31 ± 2.81	−0.99 ± 3.19	0.41	−0.24 ± 1.09	0.32 ± 1.18	0.29
	U6R	−0.39 ± 1.58	−0.26 ± 1.82	0.65	−0.43 ± 2.47	−0.05 ± 2.83	0.55	0.13 ± 1.86	0.38 ± 1.92	0.71
	U1L	0.32 ± 2.36	0.23 ± 2.91	0.81	−1.02 ± 3.27	−1.38 ± 4.01	0.30	−0.08 ± 1.09	−0.36 ± 1.03	0.57
	U1R	0.22 ± 2.23	0.27 ± 2.61	0.90	−0.86 ± 3.20	−1.09 ± 4.05	0.54	0.05 ± 1.05	−0.22 ± 1.12	0.58
	U1	0.27 ± 2.29	0.25 ± 2.74	0.95	−0.94 ± 3.23	−1.23 ± 4.03	0.42	−0.02 ± 1.07	−0.29 ± 1.07	0.58

All data are expressed as mean ± SD. We performed the pair t-test and Wilcoxon test

However, using absolute discrepancy, significant differences were found in U3L, U1L, U1R and U1 (*p* < 0.05) in anterior – posterior direction, with VSP group showing higher discrepancies than CSP group (2.14 mm – 1.01 mm, 1.97 mm – 1.18 mm, 1.85 mm – 1.14 mm, 1.92 mm – 1.13 mm, respectively) (Table 5).

**Table 5:** Comparison of the absolute values of positional changes in maxillary landmarks between P3D and actual postoperative outcomes in each group and differences between the two groups

		VSP			CSP			Different		p
		P3D	Post op	p	P3D	Post op	p	Test	Control	
X	A	0.90 ± 0.48	0.45 ± 0.35	0.02	0.66 ± 0.48	0.72 ± 0.53	0.69	0.99 ± 0.70	1.07 ± 0.73	0.79
	ANS	1.07 ± 0.54	0.43 ± 0.35	0.01	0.73 ± 0.62	0.71 ± 0.51	0.90	1.15 ± 0.65	1.06 ± 0.83	0.78
	U3L	0.73 ± 0.63	0.68 ± 0.52	0.81	0.65 ± 0.42	1.16 ± 1.14	0.21	0.79 ± 0.63	1.43 ± 1.16	0.15
	U3R	0.72 ± 0.64	1.00 ± 1.18	0.46	0.66 ± 0.41	1.18 ± 1.22	0.25	0.95 ± 0.76	1.49 ± 1.25	0.26
	U6L	0.68 ± 0.54	0.66 ± 0.52	0.95	0.83 ± 0.60	0.89 ± 0.91	0.87	0.92 ± 0.49	1.60 ± 1.17	0.11
	U6R	0.70 ± 0.54	1.15 ± 0.50	0.15	0.90 ± 0.69	0.96 ± 0.87	0.88	1.27 ± 0.61	1.57 ± 1.02	0.44
	U1L	0.82 ± 0.83	0.74 ± 0.49	0.76	0.85 ± 0.47	1.31 ± 1.30	0.30	0.89 ± 0.48	1.35 ± 1.11	0.25
	U1R	0.83 ± 0.82	0.69 ± 0.70	0.57	0.86 ± 0.47	1.43 ± 1.33	0.21	0.74 ± 0.48	1.49 ± 1.18	0.09
	U1	0.82 ± 0.82	0.69 ± 0.58	0.60	0.86 ± 0.47	1.35 ± 1.34	028	0.82 ± 0.46	1.43 ± 1.14	0.14
Y	A	2.70 ± 1.82	2.69 ± 0.85	0.99	2.61 ± 1.88	2.12 ± 1.57	0.16	1.25 ± 0.80	0.90 ± 0.63	0.29
	ANS	2.83 ± 1.96	1.99 ± 1.15	0.30	2.77 ± 1.97	2.06 ± 1.82	0.15	2.05 ± 1.70	1.66 ± 1.25	0.57
	U3L	2.70 ± 1.64	2.75 ± 1.10	0.95	2.66 ± 1.58	3.00 ± 1.30	0.39	2.14 ± 0.85	1.01 ± 0.64	0.00
	U3R	2.28 ± 0.95	2.38 ± 1.22	0.84	2.04 ± 1.61	2.19 ± 1.33	0.59	1.62 ± 1.06	0.86 ± 0.58	0.06
	U6L	2.92 ± 1.81	2.63 ± 1.00	0.67	2.89 ± 1.90	2.79 ± 1.75	0.84	1.84 ± 0.90	1.27 ± 0.85	0.16
	U6R	2.38 ± 0.89	2.66 ± 1.13	0.58	2.28 ± 1.50	2.14 ± 1.46	0.60	1.76 ± 1.58	0.97 ± 1.04	0.21
	U1L	2.35 ± 1.48	2.74 ± 1.12	0.58	2.39 ± 1.31	2.69 ± 1.32	0.51	1.97 ± 0.73	1.18 ± 0.70	0.02
	U1R	2.24 ± 1.30	2.57 ± 1.19	0.61	2.20 ± 1.36	2.51 ± 1.33	0.49	1.85 ± 0.78	1.14 ± 0.64	0.04
	U1	2.26 ± 1.45	2.66 ± 1.17	0.56	2.30 ± 1.32	2.59 ± 1.31	0.51	1.92 ± 0.75	1.13 ± 0.70	0.03
Z	A	1.79 ± 1.05	2.53 ± 1.63	0.03	2.53 ± 1.85	2.81 ± 2.31	0.44	1.15 ± 0.58	0.81 ± 0.77	0.27
	ANS	1.83 ± 1.07	2.27 ± 1.31	0.15	2.56 ± 1.89	2.80 ± 1.85	0.52	0.93 ± 0.88	0.92 ± 0.93	0.98
	U3L	1.96 ± 1.35	2.02 ± 1.41	0.77	2.54 ± 2.09	2.95 ± 2.56	0.18	0.82 ± 0.68	0.72 ± 0.64	0.73
	U3R	1.63 ± 0.62	2.01 ± 1.11	0.24	2.32 ± 1.53	2.99 ± 2.10	0.15	0.87 ± 0.77	1.23 ± 0.82	0.33
	U6L	1.98 ± 1.41	1.45 ± 1.56	0.09	2.10 ± 2.21	2.51 ± 2.07	0.21	0.89 ± 0.62	1.04 ± 0.56	0.58
	U6R	1.34 ± 0.81	1.54 ± 0.87	0.38	1.73 ± 1.73	2.31 ± 1.45	0.33	0.74 ± 0.39	1.53 ± 1.11	0.06
	U1L	1.99 ± 1.13	2.35 ± 1.55	0.26	2.68 ± 1.96	3.26 ± 2.52	0.07	0.77 ± 0.74	0.71 ± 0.80	0.87
	U1R	1.92 ± 0.97	2.15 ± 1.32	0.44	2.66 ± 1.80	3.31 ± 2.36	0.05	0.76 ± 0.68	0.81 ± 0.76	0.88
	U1	1.93 ± 1.09	2.25 ± 1.41	0.30	2.66 ± 1.90	3.29 ± 2.43	0.05	0.74 ± 0.73	0.75 ± 0.78	0.97

All data are expressed as mean ± SD. We performed the independent t-test and Mann–Whitney test

### Cast model surgery and VSP

The comparison of virtual surgical planning (VSP) and model surgery in anterior–posterior (Y) and vertical (Z) positioning showed no statistically significant differences (*p* > 0.05) in most landmarks.

Minor variations were observed in the U6L landmark, exhibiting a trend toward under-correction in model surgery in Y-axis (anterior- posterior) with 1.73 and 1.31 mm (*p* < 0.05); and the Z-axis (vertical positioning) with −1.31 mm and −0.80 mm (*p* < 0.05). For absolute errors, all of values were comparable between VSP and model surgery (*p* > 0.05) (Table 6).

**Table 6:** Comparison of the positional changes in maxillary landmarks between P3D and PCM and differences between the two groups (*n* = 10)

		Direction			Asolute			Different		
		P3D	PCM	p	P3D	PCM	p	VSP	PCM	p
Y	U3L	1.62 ± 2.73	1.51 ± 2.43	0.59	2.24 ± 1.68	2.37 ± 1.47	0.76	0.42 ± 1.16	0.10 ± 2.23	0.67
	U3R	0.93 ± 2.50	0.74 ± 2.67	0.45	2.01 ± 1.64	2.33 ± 1.30	0.19	0.30 ± 1.03	0.07 ± 2.08	0.76
	U6L	1.73 ± 3.09	1.31 ± 2.86	0.03	2.37 ± 1.90	2.57 ± 1.67	0.70	0.10 ± 1.59	0.00 ± 2.19	0.90
	U6R	0.66 ± 2.75	0.76 ± 2.92	0.71	2.11 ± 1.66	2.48 ± 1.53	0.24	0.30 ± 1.43	−0.30 ± 2.69	0.46
	U1L	1.38 ± 2.44	1.50 ± 2.37	0.29	2.14 ± 1.51	2.37 ± 1.36	0.44	0.33 ± 1.38	−0.21 ± 2.18	0.46
	U1R	1.21 ± 2.37	1.25 ± 2.31	0.82	2.06 ± 1.52	2.11 ± 1.47	0.82	0.38 ± 1.31	−0.07 ± 2.40	0.56
Z	U3L	−1.23 ± 3.14	−1.25 ± 3.09	0.92	2.37 ± 2.23	2.55 ± 2.00	0.30	−0.16 ± 0.98	−0.43 ± 1.71	0.40
	U3R	−0.62 ± 2.81	−0.75 ± 2.62	0.47	2.23 ± 1.65	2.14 ± 1.54	0.61	0.01 ± 1.54	−0.21 ± 2.27	0.56
	U6L	−1.31 ± 2.81	−0.80 ± 2.85	0.00	1.96 ± 2.31	2.04 ± 2.05	0.76	0.32 ± 1.18	−0.31 ± 1.33	0.00
	U6R	−0.43 ± 2.47	−0.54 ± 2.82	0.79	1.72 ± 1.75	2.12 ± 1.80	0.32	0.38 ± 1.92	0.28 ± 1.87	0.71
	U1L	−1.02 ± 3.27	−1.13 ± 3.23	0.32	2.52 ± 2.12	2.72 ± 1.91	0.30	−0.36 ± 1.03	−0.66 ± 1.91	0.38
	U1R	−0.86 ± 3.20	−0.59 ± 3.43	0.59	2.51 ± 1.96	2.86 ± 1.75	0.17	−0.22 ± 1.12	−0.90 ± 3.06	0.39

All data are expressed as mean ± SD. We performed the pair t-test and Wilcoxon test

## Discussion

This study aimed to compare the accuracy of VSP and CSP in bimaxillary orthognathic surgery, focusing on differences in positional accuracy across the X (medial–lateral), Y (anterior–posterior), and Z (superior-inferior) axes. The comparison of VSP and CSP revealed that the clinically achieved predictability of both methods was similar.

VSP and CSP demonstrated no significant differences between the planned and postoperative outcomes across all dimensions. When comparing VSP predictions with actual postoperative outcomes, no statistically significant differences were observed across all three axes. However, assessment of absolute discrepancies revealed that VSP exhibited greater deviations than CSP in certain maxillary landmarks, particularly in the anterior–posterior direction (Y-axis) at U3L, U1L, U1R, and U1 (*p* < 0.05), where VSP showed larger errors compared to CSP.

### Comparison between 2 and 3D planning and actual postoperative outcomes

Our results demonstrated that 3D planning significantly differed from 2D planning at key maxillary landmarks, particularly in the Y-axis (anterior–posterior direction). Notably, U1L, U1R, U3L, U3R, and U6R showed significant differences indicating that VSP provided a more refined and accurate surgical plan compared to CSP. These findings are consistent with Ho [8], who also reported discrepancies in landmark positioning along the Y-axis. A possible explanation for this difference is that 3D planning allows for better visualization of yaw rotation, which are often not as evident in 2D planning methods. As a result, yaw adjustments are systematically incorporated in VSP to achieve optimal maxillary and mandibular symmetry. This continuous refinement in 3D surgical planning could contribute to the observed differences in anterior–posterior positioning of maxillary landmarks compared to CSP.

Our findings demonstrated that postoperative outcomes did not significantly differ from the 3D surgical plan, regardless of whether the surgical splints were fabricated using manual (CSP) or 3D-printed (VSP) techniques. This result highlights that the 3D surgical plan itself is the key determinant of surgical accuracy. 3D virtual planning provided a clear visualization of key surgical parameters, including bone contact points, bony gaps, and precise skeletal movements on the simulated surgical model. This enhanced preoperative understanding, combined with the use of surgical splints, enabled accurate replication of the digital treatment plan intraoperatively. The integration of both surgical guides splint and detailed 3D visualization ultimately facilitated precise execution of the surgical plan, contributing to optimal patient outcomes.

Comparison of the discrepancies between VSP and CSP relative to the planned surgical outcomes revealed no statistically significant differences between the two groups. This suggests that both VSP and CSP offer comparable accuracy in terms of overall surgical outcomes. However, analysis of absolute values of discrepancies revealed the VSP group exhibited larger deviations than the CSP group, particularly at U3L, U1L, U1R, and U1. One possible explanation for this discrepancy could be the difference in splint thickness and material properties between the two methods [9]. The VSP splints were thinner and had lower flexural strength compared to the CSP splints, which may have affected their resistance to deformation during surgery. Due to the manufacturing process, CSP splints tend to be thicker, which may have improved their stability intraoperatively [7, 9]. However, if the surgical splint is too thick, especially when the depth of the cusps embedded in the splint exceeds 3 mm, it may reduce surgical accuracy due to premature contacts between the teeth and the splint [17].

In our study, most patients presented with skeletal Class III deformities requiring anterior and inferior maxillary repositioning. Such movements often result in occlusal overlaps between the upper and lower jaws, requiring a vertical mandibular opening during virtual surgical planning to fabricate a viable intermediate splint. Consequently, the determination of a condylar hinge axis becomes a critical step. In VSP, this axis is commonly defined at the posterosuperior point of the condyle. However, existing literature reveals considerable interindividual variability in the instantaneous center of rotation (ICR), which shifts dynamically during mandibular movement. Previous studies have shown that errors in defining this rotational axis can lead to significant discrepancies in maxillary positioning, especially in the sagittal plane, due to inaccurate simulation of mandibular autorotation [6]. Therefore, precise hinge axis determination remains a critical focus for improving the accuracy of virtual planning in orthognathic surgery.

Another contributing factor may be inaccuracies in centric relation (CR) registration. Our protocol used wax bite records to define CR for 3D simulation. However, the concept of CR remains controversial. While the Glossary of Prosthodontic Terms defines it as the most anterior-superior condylar position, orthognathic surgeons often adopt a posterior-superior manipulation, termeds retruded contact position. Misalignment between clinical practice and theoretical definitions may result in unstable mandibular positioning, particularly in patients with complex occlusal discrepancies. This introduces spatial errors that cascade into maxillary misalignment during intermediate splint design and surgical execution [3].

Despite technological advances in 3D printing, reliance on splint-only transfer remains a limiting factor. A recent study reported vertical errors up to 5 mm in anterior maxillary positioning when using 3D-printed splints alone, highlighting the impact of surgical technique, lack of rigid reference points, condylar positioning variability, and bony interference during osteotomy [11].

Surgical sequencing may impact accuracy. While patient-specific osteosynthesis (PSO) systems typically offer higher precision, evidence shows that maxilla-first sequencing results in greater deviations compared to mandible-first protocols (1.8 mm vs. 1.0 mm; *p* = *0.008*) and 40.5% of the cases had a deviation of > 2 mm in any direction at the upper incisor point, due to increased reliance on stable condylar seating during maxillary repositioning [16]. As our study relied solely on occlusal splints and manually bent fixation plates—without PSO—the likelihood of positional inaccuracies may be inherently higher.

Moreover, recent comparative studies highlighte the limitations of occlusal splints, whether manually fabricated conventional resin occlusal splint (CROS) or 3D-printed digital occlusal splint (DOS) [5]. Quantitative analysis of maxillary repositioning accuracy revealed mean deviations of 2.55 ± 0.95 mm for CROS, 2.15 ± 1.12 mm for DOS, and a significantly lower at 1.17 ± 0.66 mm for the digital template group (*P* < 0.001 vs. CROS; *P* = 0.001 vs. DOS). These findings highlight that, despite improvements in fabrication, occlusal splints—whether digital or conventional—remain dependent on mandibular positioning and lack vertical control of the maxilla. Notably, no significant difference was found between CROS and DOS, confirming that the core limitation lies in the splint-based technique itself rather than the fabrication method. In contrast, digital templates allowed maxillary positioning independent of mandibular positioning, leading to superior accuracy.

Further research is needed to assess the impact of splint thickness, condylar rotation modeling, and material properties on surgical accuracy. Additionally, splint-less approaches using customized titanium plates and cutting guides could serve as an alternative to reduce these discrepancies and improve vertical control.

While VSP improves preoperative visualization and surgical planning, critical technical factors—including hinge axis definition, CR registration, splint stability, and surgical sequence—continue to influence the final surgical outcome. To improve accuracy, future developments in VSP should prioritize dynamic mandibular modeling, standardized CR determination methods, and transition toward template-based or splint-less protocols for improved intraoperative control and reproducibility.

### Comparison of VSP and model surgery

Comparison of VSP and model surgery, demonstrated no statistically significant differences for most maxillary landmarks, indicating that both methods provide comparable predictive accuracy. However, a trend toward under-correction in model surgery was observed at U6L in the Y-axis and Z-axis. Song et al. [13] also reported differences between cast model surgery and 3D planning in the Y-axis and Z-axis at the maxillary first molar position. These findings suggest that manual model surgery may introduce minor inaccuracies in vertical and anterior–posterior positioning.

Our results highlight the superiority of VSP over CSP for preoperative planning due to its improved precision and ability to simulate complex surgical movements in three dimensions. However, despite its advantages, VSP is not entirely error-free, particularly in anterior–posterior positioning. These discrepancies could be attributed to differences in intraoperative execution, soft tissue influences, and the complexity of translating digital plans into surgical practice.

The findings also emphasize that while VSP and model surgery yield comparable results, minor deviations in model surgery may still impact final outcomes, particularly in vertical and anterior–posterior positioning.

### Limitations and future directions

This study has several limitations. Most notably, the relatively small sample size limits the statistical power and generalizability of the findings. While the randomized controlled design strengthens the internal validity, the number of cases remains insufficient to draw definitive clinical conclusions. As such, this study serves as a pilot investigation that provides foundational data for larger-scale trials. Additionally, the analysis was limited to immediate postoperative outcomes, without evaluation of long-term skeletal stability. Future research should incorporate larger cohorts and longitudinal follow-up to comprehensively assess the clinical efficacy and durability of both VSP and CSP approaches.

## Conclusion

This study confirms that VSP provides greater accuracy in bimaxillary orthognathic at the planning stage. However, VSP exhibited slightly greater discrepancies in anterior–posterior accuracy compared to CSP, suggesting that intraoperative factors still influence final outcomes.

VSP demonstrated high predictive accuracy, with no significant differences between planned (P3D) and actual postoperative outcomes, reinforcing its reliability as a surgical planning tool. When comparing VSP and model surgery, both methods yielded comparable accuracy, but model surgery showed a slight trend toward undercorrection in certain vertical and anterior–posterior positions.

Overall, VSP should be considered the preferred planning method for bimaxillary orthognathic surgery, given its superior precision, reliability, and ability to reduce intraoperative adjustments. However, further studies with larger sample sizes and long-term follow-ups are necessary to confirm its long-term stability and clinical benefits.

## Data Availability

No datasets were generated or analysed during the current study.

## Abbreviations

VSP: Virtual surgical planning
CSP: Conventional surgical planning
CT: Computed tomography
RCT: Randomized controlled trial
P2D: 2D planning
P3D: 3D planning
PCM: Planning cast model
BSSO: Bilateral sagittal split osteotomy
IMF: Intermaxillary fixation
ICC: Intraclass correlation coefficient
CR: Centric relation
PSO: Patient-specific osteosynthesis
CROS: Conventional resin occlusal splint
DOS: Digital occlusal splint
